# A Model and Quantitative Framework for Evaluating Iterative Steganography

**DOI:** 10.3390/e26121130

**Published:** 2024-12-23

**Authors:** Marcin Pery, Robert Waszkowski

**Affiliations:** Institute of Computer and Information Systems, Faculty of Cybernetics, Military University of Technology, 00-908 Warszawa, Poland; marcin.pery@wat.edu.pl

**Keywords:** steganography, information quantification, iterative steganography, Incremental Information Function (IIF), steganographic modeling, information hiding

## Abstract

This study presents a detailed characterization of iterative steganography, a unique class of information-hiding techniques, and proposes a formal mathematical model for their description. A novel quantitative measure, the Incremental Information Function (IIF), is introduced to evaluate the process of information gain in iterative steganographic methods. The IIF offers a comprehensive framework for analyzing the step-by-step process of embedding information into a cover medium, focusing on the cumulative effects of each iteration in the encoding and decoding cycles. The practical application and efficacy of the proposed method are demonstrated using detailed case studies in video steganography. These examples highlight the utility of the IIF in delineating the properties and characteristics of iterative steganographic techniques. The findings reveal that the IIF effectively captures the incremental nature of information embedding and serves as a valuable tool for assessing the robustness and capacity of steganographic systems. This research provides significant insights into the field of information hiding, particularly in the development and evaluation of advanced steganographic methods. The IIF emerges as an innovative and practical analytical tool for researchers, offering a quantitative approach to understanding and optimizing iterative steganographic techniques.

## 1. Introduction

Steganography is recognized as a well-established field of study [1] situated within the broader framework of information theory [2]. The primary focus of this discipline is the development and analysis of methods and techniques for concealing information within overt covers so that third parties remain unaware of the existence of the hidden data [3].

### 1.1. Steganography

A wide range of steganographic techniques has been extensively documented in the literature [4]. The primary goal of these methods is to encode information within a digital cover medium, producing a steganogram that closely resembles the original cover. This resemblance is intended to make the detection of embedded messages highly challenging or practically infeasible [5]. The choice of a suitable steganographic technique depends on several factors [6], including the type of the cover medium, which may consist of text files, digital images, audio streams, video sequences, or network transmissions. Moreover, the selection is influenced by specific requirements regarding the desired properties of the steganographic method, such as capacity, robustness, and undetectability [7].

In recent years, one of the most actively investigated directions in research on steganographic techniques has been the domain of machine learning, including the use of neural networks. One of the latest examples of a novel approach advancing steganographic techniques can be found in the paper [8], which introduces an innovative invisible and robust watermarking model for digital images. It employs hierarchical residual fusion and multi-scale convolution techniques to embed imperceptible yet resilient watermarks. The proposed neural network-based approach aims to balance image quality preservation with watermark durability against various image manipulations, likely demonstrating improved PSNR, SSIM, and resistance to common image transformations. Another example can be found in [9]. This paper introduces a novel dual color image watermarking scheme utilizing quaternion fractional-order orthogonal Fourier–Mellin moments and least square support vector regression for geometric correction. The method employs advanced techniques including 3D sine map encryption, lifting wavelet transform, and quaternion discrete Fourier transform to enhance security and robustness. Experimental results demonstrate high imperceptibility and strong robustness against various attacks, representing a significant advancement in color image watermarking and copyright protection.

Although the various approaches found in the literature are highly valuable in multiple respects and advance the field of steganography in several promising directions, the literature lacks any references directly addressing the core topic of our work, i.e., the formalization and quantification of the fundamental properties of iterative steganography.

We utilize three fundamental metrics to define the core properties of steganographic techniques. These metrics are often interdependent and involve trade-offs in steganographic system design. Achieving the optimal balance between these properties depends on the specific application requirements and the anticipated threats to the steganographic communication channel:

- Undetectability: this metric evaluates a steganographic technique’s ability to conceal information within a steganogram so that its presence remains imperceptible to both human perception and statistical detection methods [10]. High undetectability signifies that the alterations introduced into the cover medium are minimally noticeable and resistant to detection [11],
- Capacity: this metric measures the amount of information that can be embedded within a digital cover medium without causing noticeable degradation in its quality. Capacity depends on the characteristics of the cover medium and the chosen steganographic technique. While higher capacity enables greater information concealment, it may reduce undetectability by increasing the likelihood of detection [12],
- Robustness: this metric assesses a steganographic technique’s resilience in preserving embedded information when subjected to various disruptions or transformations, such as compression, format conversion, filtering, rotation, or scaling. Higher robustness ensures that the embedded message is more likely to remain intact or that a larger portion of the original information is retained after such modifications [13].

A typical steganographic method is characterized by two core functions: an encoding function, which embeds the message within the cover medium, and a decoding function, which retrieves the embedded message from the steganogram:

- Encoding function—maps a given cover object and a message to a steganogram. The encoding process embeds the message within the cover medium while minimizing perceptible alterations to the cover medium,
- Decoding function—extracts the embedded message from a steganogram. It is designed to accurately retrieve the message without requiring access to the original cover object. Notably, the decoding function can also be considered as an integral part of the steganalysis process, as it helps verify the presence and content of hidden information [14].

The performance of these functions is assessed based on their ability to preserve the cover medium’s integrity, maximize the capacity for concealed information, and withstand detection by steganalysis techniques. Designing these functions often requires managing significant trade-offs between undetectability, capacity, and robustness, which are crucial for the effectiveness of steganographic methods.

### 1.2. Iterative Steganography

Iterative steganography is a unique approach to information hiding, distinct from traditional techniques that encode data within a single, complete unit, such as individual images [15] or text documents [16]. This method is designed for covers with an inherent iterative structure, consisting of multiple repeated elements over time. In iterative steganography, information is encoded incrementally, leveraging the cover’s natural division into homogeneous segments. The full retrieval of the hidden message is only possible after multiple iterations.

Prominent examples of iterative steganography include video steganography [17], where individual video frames naturally serve as iterations for encoding information, and network steganography [18], which utilizes repeated network packet calls as iterations in the steganographic process.

A key feature of iterative steganography is its temporal dimension, which organizes consecutive iterations over time. Any repetitive phenomenon with intervals of information transmission can serve as an effective cover for iterative steganography.

In steganographic methods based on iterative algorithms, each iteration involves two counteracting processes:

- Information increment: arising from the decoding of data embedded within the steganogram,
- Information degradation: caused by transformations or interference affecting the steganogram.

The successful retrieval of hidden information depends on the rate of information gain surpassing the rate of degradation, and the decoded information reaching a critical threshold necessary for complete message recovery.

This study seeks to develop a formal method for quantifying the balance between information gain and loss in each iteration of iterative steganographic techniques. The aim is to determine whether the incremental accumulation of information over successive decoding iterations provides meaningful insights into the characteristics and performance of these methods. By introducing the Incremental Information Function (IIF), this research establishes a comprehensive framework for analyzing information increments in iterative steganographic processes. In contrast to traditional steganography, which focuses on single instance encoding, iterative methods require a distinct approach to understanding how information is progressively embedded and extracted over multiple iterations. Practical examples are presented to illustrate the applicability of the proposed method, providing a robust tool for evaluating and optimizing the efficiency, robustness, and capacity of iterative steganographic systems.

## 2. A Model for Iterative Steganography

For this study, a single *iteration* is defined as a binary vector that is characteristic of specific type of iterative cover. For instance, in video files, this might correspond to a single video frame; for audio files, it could be a time frame used for calculating Fast Fourier Transform (FFT) frequency components; and for network transmissions, it may represent a data packet from a specific network protocol along with associated timing parameters. Depending on the context and type of cover, individual bits within a single iteration can hold varying significance and semantic interpretation. The core assumption of iterative steganography is that, for a specific type of iterative cover, all iterations are homogeneous—identical in their structure, properties, and characteristics critical to the steganographic encoding and decoding algorithms.

### 2.1. Iterative Cover

The *iterative cover* $c^{i}$ is defined as an i-element vector (refer to Equation (1)).

(1) $$c^{i}=\left[c_{1},\;c_{2},...,c_{i}\right]$$

where $c_{i}$—the i-th iteration of the $c^{i}$, corresponding to the i-th encoding step.

An iterative cover can be regarded as a vector of iteration, constructed by sequentially appending each iteration in order, from the first to the last (refer to Equation (2)).

(2) $$\underset{i\in \left\{1...Z\right\}}{⋀}c^{i}=\left\{\begin{array}{c} \left[c_{1}\right],\;\;\;\;\;\;\;\;\;\;\;\;\;\;\;\;\;\;\;\;\;\;\;\;\;\;i=1\\ c^{i-1}⨁c_{i},\;\;\;\;\;\;\;i\in \left\{2...Z\right\} \end{array}\right.\;\underset{i\in \left\{1...Z\right\}}{⋀}c_{i}\in I,\;\;\;\;\;\;{I=\left\{0,1\right\}}^{*}\;\underset{i\in \left\{1...Z\right\}}{⋀}c^{i}\in C,\;\;\;\;\;\;\;\;C=I^{*}\;Z\in N$$

where

Z—the number of iterations, equivalent to the number of encoding steps;
I—the set of all iterations, defined as the set of all possible finite binary vectors;
C—the set of all iterative covers.

### 2.2. Message

The *message*, m, is defined as a vector of L bits (refer to Equation (3)).

(3) $$m=\left[m_{1},\;m_{2},\;\ldots ,\;{m_{k},...,m}_{L}\right]\;\underset{k\in \left\{1...Z\right\}}{⋀}m_{k}\in \left\{0,\;1\right\}\;L\in N,\;m\in M,{M=\left\{0,\;1\right\}}^{*}$$

where

L—the number of bits in the message, m (message length);
$m_{k}$—the k-th bit of the message, m;
M—the set of all messages.

Let us define $m\left(1\right)$ as the number of 1’s in message m (refer to Equation (4)).

(4) $$m\left(1\right)=\sum_{k=1}^{L}1_{\left\{m_{k}=1\right\}}$$

Let us define $m\left(0\right)$ as the number of 0’s in message m (refer to Equation (5)).

(5) $$m\left(0\right)=\sum_{k=1}^{L}1_{\left\{m_{k}=0\right\}}$$

$$m\left(0\right)+m\left(1\right)=L$$

### 2.3. Iterative Steganogram

The *iterative steganogram*, $s^{i}$, is defined analogously to the iterative cover, $c^{i}$, as an i-element vector (refer to Equation (6)).

(6) $$s^{i}=\left[s_{1},\;s_{2},...,s_{i}\right]$$

where $s_{i}$—the i-th iteration of the $s^{i}$, corresponding to the i-th decoding step.

An iterative steganogram can be regarded as a vector, constructed by sequentially appending each iteration in order, from the first to the last (refer to Equation (7)).

(7) $$\underset{i\in \left\{1...Z\right\}}{⋀}s^{i}=\left\{\begin{array}{c} \left[s_{1}\right],\;\;\;\;\;\;\;\;\;\;\;\;\;\;\;\;\;\;\;\;\;\;\;\;\;\;i=1\\ s^{i-1}⨁s_{i},\;\;\;\;\;\;\;i\in \left\{2...Z\right\} \end{array}\right.\;\underset{i\in \left\{1...Z\right\}}{⋀}s_{i}\in I\;\underset{i\in \left\{1...Z\right\}}{⋀}s^{i}\in S,\;\;S=I^{*}\;Z\in N$$

where

Z—the number of iterations, equivalent to the number of decoding steps;
S—the set of all iterative steganograms.

### 2.4. Coding Indices

From the set of all possible indices of the iterative cover and the iterative steganogram, we select a subset, referred to as the *coding indices* (refer to Equation (8)).

(8) $$Ι\subseteq \left\{2...Z\right\}\;Ι\in 2^{\left\{2...Z\right\}}$$

where Ι—the set of coding indices.

### 2.5. Encoding and Decoding Iterations

From the set of all iterations in the cover and the set of all iterations in the steganogram, we select subsets using coding indices, referred to as the *encoding* and *decoding iterations* (refer to Equations (9) and (10)).

(9) $$c^{I}=\left\{c_{i}:i\in I\right\}$$

(10) $$s^{I}=\left\{s_{i}:i\in I\right\}$$

where

$c^{I}$—the encoding iterations, represented as a subset of all cover iterations and selected using the coding indices, I;
$s^{I}$—the decoding iterations, represented as a subset of all steganogram iterations and selected using the coding indices, I.

### 2.6. Encoding Function

We assume that in each iteration, the entire message, m, is encoded. However, it is also possible to consider scenarios where the message, m, is divided into disjoint parts, with each part treated as a separated message subjected to independent encoding.

First, we define the *encoding function*, f′, which encodes the message, m, in the i-th iteration of the cover (refer to Equation (11)).

(11) $$f^{\prime }:I\times M\to I$$

$$\underset{i\in \left\{1...Z\right\}}{⋀}s_{i}:=f\prime \left(c_{i},m\right)$$

where m—the message to be encoded in the i-th iteration.

The *iterative encoding function*, f, is defined as the function that encodes the message, m, in the cover (refer to Equation (12)).

(12) f:C×M→S

$$\underset{i\in \left\{1...Z\right\}}{⋀}s^{i}:=f\left(c^{i},m\right)$$

where

$c^{i}$—the cover composed of i iterations;
$s^{i}$—the steganogram created by the function f through encoding the message m into the cover $c^{i}$.

An iterative steganogram can be regarded as a vector formed by sequentially appending the values of f′ calculated for consecutive iterations (refer to Equation (13)).

(13) $$\underset{i\in \left\{1...Z\right\}}{⋀}f\left(c^{i},m\right)=\left\{\begin{array}{c} \left[c_{1}\right],\;\;\;\;\;\;\;\;\;\;\;\;\;\;\;\;\;\;\;\;\;\;\;\;\;\;\;\;\;\;\;\;\;\;\;\;\;\;\;\;\;\;\;i=1\\ f\left(c^{i-1},m\right)⨁c_{i},\;\;\;\;\;\;\;\;\;\;\;\;\;\;\;\;\;\;\;\;\;\;i\notin I\\ f\left(c^{i-1},m\right)⨁f\prime \left(c_{i},m\right),\;\;\;\;\;\;\;\;\;\;i\in I \end{array}\right.$$

### 2.7. Decoding Function

The *iterative decoding function*, $f^{-1}$, is defined as the function that maps a decoded message, $m^{-1}$, to a given steganogram, $s^{i}$ (refer to Equation (14)).

The decoding function produces the decoded message, $m^{-1}$, which, like the original message, m, is a binary vector from the set {0, 1}.

The purpose of the decoding function is to recover the information encoded in the steganogram, $s^{i}$. If the decoded message, $m^{-1}$, matches to the original message, m, the goal is fully achieved. However, if $m\neq m^{-1}$, the ability to accurately interpret the information from the decoded message depends primarily on two factors: (1) the amount of preserved information in the message, $m^{-1}$, which can be evaluated, for example, using a distance measure between the decoded and the original message; and (2) the properties of the steganographic technique employed, including the degree of redundancy incorporated during the encoding process.

(14) $$f^{-1}:S\to M$$

$$m^{-1}:=f^{-1}\left(s^{i}\right)\;m^{-1}\in M$$

where $m^{-1}$—the message decoded by the function $f^{-1}$ from the steganogram $s^{i}$.

## 3. The Proposed Method

### 3.1. IIF (Incremental Information Function)

The definition of the Incremental Information Function (IIF) is inspired by the concept of the Bit Error Rate (BER), which measures the ratio of incorrectly encoded bits to the total number of encoded bits in a message [19]. A lower BER indicates less information loss in the encoded message and higher robustness. In contrast, for the IIF, a higher function value signifies a greater amount of information decoded from the steganogram. The details of the relationship between the IIF and the BER are described in Section 3.5.

First, we define the auxiliary function ${IIF}_{\left(orig,dec\right)}$ (refer to Equation (15)), which assigns to a given steganogram, $s^{i}$, and message, m, the relative number of occurrences of decoded bits with the value dec that had the value orig in the original message, m.

(15) $${IIF}_{\left(orig,dec\right)}:S\times M\to R^{+}$$

$$\underset{\begin{array}{c} orig\in \left\{0,1\right\}\\ dec\in \left\{0,1\right\} \end{array}}{⋀}\underset{i\in \left\{1...Z\right\}}{⋀}{IIF}_{\left(orig,dec\right)}\left(s^{i},\;m\right)=\sum_{k=1}^{L}1_{\left\{m_{k}=orig\wedge {f^{-1}\left(s^{i}\right)}_{k}=dec\right\}}/m\left(orig\right)\;\underset{i\in \left\{1...Z\right\}}{⋀}\underset{orig\in \left\{0,1\right\}}{⋀}\sum_{dec\in \left\{0,1\right\}}{IIF}_{\left(orig,dec\right)}\left(s^{i},\;m\right)=1$$

where

orig—the value of a bit in the original message m;
dec—the value of a bit in the decoded message $m^{-1}$;
m(orig)—the number of orig-bites in the message m (either m(0) or m(1));
$f^{-1}$—the decoding function;
m—the message encoded by function f into the cover $c^{i}$.

The IIF function (refer to Equation (16)) is defined as a weighted sum of the functions ${IIF}_{\left(0,0\right)}$ and ${IIF}_{\left(1,1\right)}$, where the weights are proportional to the number of bits with values 0 and 1 in the original message, m. This represents the proportion of correctly decoded bits in the message, $m^{-1}$.

(16) $$IIF:S\times M\to \langle 0,1\rangle$$

$$\underset{i\in \left\{1...Z\right\}}{⋀}IIF\left(s^{i},\;m\right)={IIF}_{\left(0,0\right)}\left(s^{i},\;m\right)\cdot \frac{m\left(0\right)}{L}+{IIF}_{\left(1,1\right)}\left(s^{i},\;m\right)\cdot \frac{m(1)}{L}=\sum_{k=1}^{L}1_{\left\{m_{k}={f^{-1}\left(s^{i}\right)}_{k}\right\}}/L$$

where

m—the message decoded by the function $f^{-1}$ from the steganogram, $s^{i}$;
m(0)—the number of 0’s in the message, m;
m(1)—the number of 1’s in the message, m.

In Equation (16), which defines the IIF, it is also possible to apply different weights, particularly in scenarios where the steganographic method processes 0’s and 1’s differently. For instance, in a hypothetical case where 0’s are significantly less important than 1’s, the weight for ${IIF}_{\left(0,0\right)}$ might not be proportional to $m\left(0\right)$ but could instead be, for example, one-tenth of the weight for the ${IIF}_{\left(1,1\right)}$. Additionally, in some specific steganographic techniques, the values of the ${IIF}_{\left(0,1\right)}$ and the ${IIF}_{\left(1,0\right)}$ can be incorporated into the definition of the IIF function as “penalties” for errors in training the decoding algorithm. It should be noted that the ${IIF}_{\left(0,1\right)}=1-{IIF}_{\left(0,0\right)}$ and ${IIF}_{\left(1,0\right)}=1-{IIF}_{\left(1,1\right)}$.

### 3.2. Properties of the IIF

The behavior of the Incremental Information Function (IIF) depends on the specific implementation of the encoding and decoding functions. Based on conducted experiments, this behavior appears to be more significantly influenced by the form of the decoding function, which relies on critical assumptions from the initial iterations—for instance, regarding the initial bit values of the decoded message. Subsequent iterations contribute additional increments of information, progressively building the overall data and ultimately determining whether the entire message is successfully decoded.

Drawing on the adopted definition and the nature of the IIF, the following key characteristics can be emphasized:

1. **Range of values**: the values of the IIF are contained within the closed interval [0, 1], with values between approximately 0.5 and 1 being of particular importance. Smaller values of the IIF indicate a higher presence of noise, while larger values reflect a greater degree of encoded information.
2. **Monotonicity**: for a properly functioning steganographic technique, featuring a well-defined encoding and decoding algorithm, the IIF is expected to remain non-decreasing, except for rare, isolated cases where the iterative steganogram exhibits non-uniformity, resulting in temporary disruptions to monotonicity. Each successive iteration of the decoding algorithm should either increase the amount of information in the decoded message, or at worst, maintain it without any decrease.
3. **Asymptotic behavior**: the IIF demonstrates asymptotic tendencies, approaching the maximum achievable level of information that can be decoded from a given steganogram, subject to specified noise conditions and the parameter settings of the encoding algorithm. In most cases, this value will not reach 1, which would represent the complete decoding of all information encoded in the original steganogram. However, it is expected to represent a sufficiently significant portion of the encoded information, enabling the recipient to decode the message content.

### 3.3. Characteristic Values

The characteristic value, ${chIIF}^{s}$, is defined as the smallest value of the IIF function at which the information extracted from the steganogram, s, is sufficient to decode the message, m. The level of ${chIIF}^{s}$ serves as a defining feature that strongly characterizes a specific steganographic technique.

Conversely, the characteristic value, ${chIteration}^{s}$, is defined as the first iteration at which the IIF function attains the characteristic value, ${chIIF}^{s}$ (refer to Equation (17)).

(17) $${chIIF}^{s}=IIF(s^{{chIteration}^{s}},\;m)$$

where

s—the steganogram created by encoding the message, m;
m—the message that is encoded into the steganogram, s;
${chIIF}^{s}$—the minimum value of the IIF required to successfully decode the message, m, from the steganogram, s,
${chIteration}^{s}$—the first iteration at which the IIF reaches the value of ${chIIF}^{s}$.

The characteristic value, ${chIIF}^{s},$ serves as an indicator of the robustness of a given steganographic technique and the steganogram, s, itself. A lower value of ${chIIF}^{s}$ implies that less decoded information is required to reveal the hidden message, allowing for greater tolerance to distortions or transformations in the steganogram.

Conversely, the characteristic value, ${chIteration}^{s}$, serves as an indicator of the steganogram’s capacity. A lower ${chIteration}^{s}$ value indicates that the hidden message is decoded faster, requiring fewer iterations. For iterative steganography, this translates to higher capacity, reflecting the ability to encode and decode more bits of information in less time. In the context of capacity, iterative steganography differs significantly from classical, non-iterative steganography. For unitary objects, capacity can be understood as a measure of the amount of information that can be encoded in a single instance, relative to the total information capacity of the individual steganogram. In iterative steganography, where the primary paradigm involves incremental changes in information during each iteration, capacity is instead understood as the amount of information that can be obtained per unit of time or per individual iteration. This distinction highlights why ${chIteration}^{s}$ serves as an effective indicator of information capacity in iterative steganography.

### 3.4. IIF Value Matrix

For each iteration of the decoding algorithm, a matrix is constructed (refer to Table 1) to present the results of the function ${IIF}_{\left(orig,dec\right)}$ corresponding to that iteration.

### 3.5. IIF and BER Indicator

In the context of steganography, the Bit Error Rate (BER) is a metric used to evaluate the accuracy of message retrieval from a steganogram. It quantifies the proportion of bits incorrectly decoded compared to the total number of bits in the original hidden message. For traditional, non-iterative steganography, the BER is calculated directly as the ratio of erroneous bits in the decoded message to the total number of bits in the original message. The BER does not distinguish between errors in decoding 0s and 1s, treating them equally and assigning the same weight to all errors in its formula. Using the defined functions ${IIF}_{\left(0,1\right)}$ and ${IIF}_{\left(1,0\right)}$, the BER can be represented mathematically, as shown in Equation (18).

(18) $$\underset{Z\in N}{⋀}\left(\sum_{i\in \left\{1...Z\right\}}BER\left(s_{i},\;m\right)=\left({IIF}_{\left(0,1\right)}\left(s^{Z},\;m\right)+{IIF}_{\left(1,0\right)}\left(s^{Z},\;m\right)\right)\cdot \frac{Z}{L}\right)$$

where

$BER\left(s_{i},\;m\right)$—the BER calculated for the iteration, $s_{i}$, and the message, m;
$s^{Z}$—the complete steganogram containing all Z iterations.

## 4. Results

### 4.1. Research Experiments

To investigate the Incremental Information Function (IIF), this study conducted experiments using video steganography as an example of an iterative steganography. A simple LSB (Least Significant Bit) [13] technique was employed to encode a two-dimensional black and white image in the spatial domain. The details of the method are as follows:

- The covers consist of video files treated as sequences of consecutive frames, each being an image;
- Successive iterations in which the message is encoded occur every third frame of the video files;
- The message is encoded using a steganographic method operating in the spatial domain, modifying the color values of the pixels in the video frames;
- The decoding function assumes that, from the first iteration, all bits of the message are initialized to zero. Subsequent iterations incrementally build information by setting 1’s and clearing 0’s in the message;
- The message is encoded in the form of a version 1 QR code with error correction level H, enabling the encoding of up to 72 bits in a 21 × 21 module matrix with error correction up to 30% [20], facilitating the use of automatic tools for reading the decoding message.

The steganography technique employed in the experiments encodes the subsequent bits of the message as follows:

- Each video frame is divided into pixel blocks, with the total number corresponding to the length of the message, and each block represents a single bit of the message.
- For consecutive pairs of video frames, the difference in color values between corresponding pixels is calculated;
- Encoding a bit value of 1 in a pixel block involves increasing the value of the R (red) color channel and the B (blue) color channel while simultaneously decreasing the value of the G (green) channel. Conversely, encoding a bit value of 0 involves increasing the G (green) color channel value while decreasing the R (red) and B (blue) color channel values;
- The technique adaptively selects pairs of pixels for encoding, focusing on those with minimal initial color differences between frames to maintain imperceptibility, particularly pixels where the color difference between consecutive video frames is zero;
- The values of the respective color channels are modified based on a parameter of the encoding algorithm, called the encoding level, which ranges from 1 to 5. At level 1, the adjustment affects only the least significant bit (00000001b), while at level 5, it modifies the three least significant bits (00000101b).

### 4.2. Example 1

**Figure 1:**
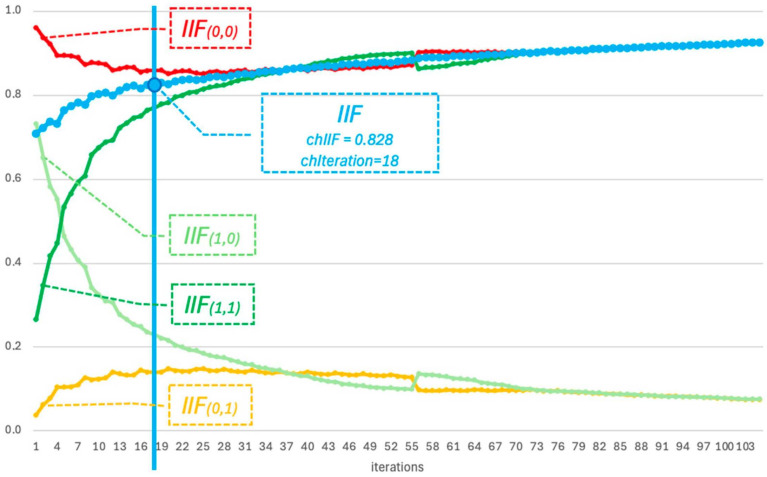
Plots of the functions *IIF*_(0,0)_, *IIF*_(1,1)_, *IIF*_(0,1)_, *IIF*_(1,0)_, and *IIF* for video steganogram No. 1.

For the first experiment, video steganogram No. 1. was selected. As a result of the decoding function’s operation, successive values of the functions ${IIF}_{\left(0,0\right)}$, ${IIF}_{\left(0,1\right)}$, ${IIF}_{\left(1,0\right)}$, ${IIF}_{\left(1,1\right)}$, and IIF were calculated. The plots of these functions are presented in Figure 1.

Figure 1 highlights, with a blue vertical line, the characteristic values, ${chIIF}^{s}$ and ${chIteration}^{s}$, which indicate the minimum threshold at which the information gain becomes sufficient for accurately decoding the hidden message.

### 4.3. Example 2

For experiment No. 2, video steganogram No. 2 was selected. This steganogram was encoded using five different encoding levels, each corresponding to a varying degree of alteration to the original cover. A higher encoding level result in greater the modification to the cover, leading to increased information capacity—the amount of information that can be encoded in the steganogram—but also reduced imperceptibility, resulting in a more noticeable difference between the steganogram and the original cover.

Table 2 illustrates the process of incremental information gain in the decoded message over the first ten iterations for video steganogram No. 2, encoded at five different encoding levels. With each decoding iteration, as the amount of information extracted from the steganogram increases, the QR code representing the message becomes progressively clearer. This corresponds to an increase in the value of the IIF function, which, after a specific number of iterations, reaches the characteristic value, ${chIIF}^{s}$, enabling the message to be fully decoded. Each cell in the table contains a black and white image of a QR code representing the decoded message, along with the corresponding IIF value for that iteration of the decoded steganogram.

Table 2 clearly demonstrates a positive correlation between the IIF and the amount of information decoded from the steganogram: the higher the IIF value, the more information can be extracted. Cells highlighted in yellow mark iterations where the amount of decoded information allowed automatic recognition of the QR code content, facilitating retrieval of the encoded message. The first yellow-highlighted cell for each encoding level determines the characteristic value, ${chIIF}^{s}$.

**Figure 2:**
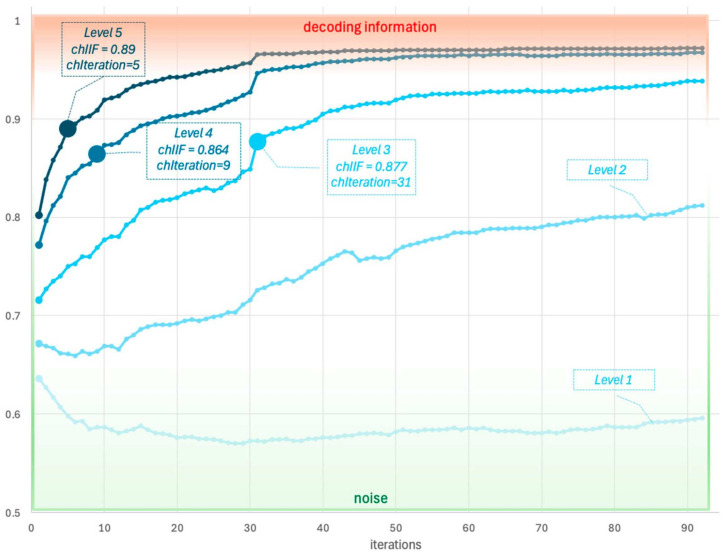
Plots of the IIF for video steganogram No. 2 encoded with 5 levels of encoding.

Figure 2, which corresponds to the results presented in Table 2, shows the plots of the IIF across the full range of decoded iterations for video steganogram No. 2, encoded at five different encoding levels. The IIF plots highlight the points where for each steganogram, the IIF attains the characteristic value, ${chIIF}^{s}$, and indicates the characteristic value, ${chIteration}^{s}$, representing the iteration at which ${chIIF}^{s}$ is reached.

Figure 2 shows the graphs of five IIF functions corresponding to five versions of a steganogram No. 2, each encoded at different encoding levels. These levels range from encoding level 1, depicted in the lightest color and representing the minimal changes introduced to the steganogram by the encoding function, to encoding level 5, depicted in the darkest color and corresponding to the highest level of changes applied. Lower encoding levels result in higher undetectability of the steganogram but come at the cost of reduced resistance to distortions and noise in the information transmitted between consecutive video frames—iterations of the iterative video steganogram.

### 4.4. Discussion

The results of first experiment, shown in Figure 1, include the plots of the functions ${IIF}_{\left(0,0\right)}$, ${IIF}_{\left(0,1\right)}$, ${IIF}_{\left(1,0\right)}$, ${IIF}_{\left(1,1\right)}$, and the overall IIF function for video steganogram No. 1. Analyzing the plots yields the following observations:

- for video steganogram No. 1, the message was successfully decoded at the 18th iteration (${chIteration}^{s}$ = 18) with an IIF value of ${chIIF}^{s}\;$= 0.828;
- the functions ${IIF}_{\left(0,0\right)}$, ${IIF}_{\left(1,1\right)}$ and the overall IIF function asymptotically converge to a maximum value close to 1.0;
- the values of ${IIF}_{\left(0,1\right)}$ and ${IIF}_{\left(1,0\right)}$ carry no significant information, in this example, as they are complementary to the values of ${IIF}_{\left(0,0\right)}$ and ${IIF}_{\left(1,1\right)}$;
- the initial value of ${IIF}_{\left(0,0\right)}$ is notably high due to a property of the decoding function, which assumes that all information bits are initialized to zero at the start of the algorithm.

The results of the second experiment, presented in Figure 2 and Table 2, show that, within the range of studied iterations, information was successfully decoded for the three highest encoding levels of video steganogram No. 2, while for the two lowest encoding levels, the IIF values remained at noise levels.

Based on the experiments, two characteristic regions of IIF values were identified: the noise region and the decoding region. The noise region corresponds to IIF values in the range of 0.5–0.7. The decoding region corresponds to IIF values in the range of 0.7–1.0, and it is within this range that the characteristic value ${chIIF}^{s}$ is found, depending on the steganographic technique used, particularly on the implementation of the decoding algorithm.

As described earlier, the properties of the IIF indicate that, in cases where hidden information can be decoded from the steganogram, the IIF increases and asymptotically approaches the maximum value specific to the steganogram. The rate of increase in the IIF is directly correlated with the encoding level, as higher encoding levels result in a greater information increment per iteration. However, if the information gain in successive iterations does not exceed the information loss associated with transitioning between iterations (e.g., due to inter-frame compression mechanisms used by video codecs), the IIF will remain static, and its values will stay within the noise region.

## 5. Conclusions

This study presents a detailed characterization and modeling framework for a class of iterative steganographic techniques. It introduces a novel method for quantifying and evaluating the performance of these techniques using the Incremental Information Function (IIF). Through a case study employing a selected video steganography method, the research illustrates the application of the IIF to analyzing the properties of iterative steganographic techniques, including the measurement of robustness and capacity by determining characteristic IIF values. This approach offers a fresh perspective on the information dynamics within iterative steganographic systems, with the potential to advance both the implementation of steganography and the effectiveness of steganalysis.

The study achieves its primary objective by developing a formal method for quantifying and analyzing information increments in iterative steganographic systems. It introduces an effective framework for assessing the efficiency and robustness of these techniques, supported by practical examples that illustrate its application to elucidate the properties of the studied steganographic methods. This advancement lays the foundation for further research and development in the field of steganography, offering a systematic and robust analytical tool to evaluate and enhance the performance of iterative techniques.

### 5.1. Theoretical Contributions

This work introduces:

- a formal mathematical model for characterizing a class of iterative steganographic methods;
- a novel quantitative method for evaluating the performance of iterative steganographic techniques, based on the proposed Incremental Information Function (IIF);
- the application of characteristic IIF values to quantify robustness and capacity metrics in iterative steganographic systems.

These contributions provide an effective framework for analyzing and optimizing iterative steganographic techniques, enhancing the theoretical foundations of this field and facilitating more precise evaluation of steganographic performance.

### 5.2. Practical Implications

The findings of this study demonstrate that the proposed method, based on the Incremental Information Function (IIF) and the analysis of its characteristic values, constitutes an effective analytical tool for investigating the properties of iterative steganographic techniques. This approach enables quantitative assessment of key performance metrics, including the robustness and capacity of iterative steganograms. The IIF-based method provides a systematic framework for evaluating and comparing different iterative steganographic algorithms, with the potential to optimize steganographic systems and improve the effectiveness of steganalysis techniques.

### 5.3. Future Research

The following avenues for further investigation are proposed:

- extension of IIF application: explore the utilization of the Incremental Information Function (IIF) in diverse iterative steganography methods beyond video steganography, such as network steganography protocols;
- universal IIF characteristics: examine the potential existence of universal characteristic values (chIIF) of the IIF that may describe and differentiate various iterative steganography techniques, including multiple video steganography methods;
- IIF in steganalysis: investigate the potential applications of IIF properties in steganalysis processes for the detection and analysis of steganographic content, potentially enhancing the efficacy of current steganalysis techniques.

These proposed research directions aim to expand the applicability and deepen the understanding of the IIF methodology in the broader context of steganography and steganalysis.

## Figures and Tables

**Table 1:** Values of ${IIF}_{\left(orig,dec\right)}$ for i-th iteration of decoding algorithm.

		**Values of Bits in the Original Message** m	
		**0**	**1**
**Values of Bits in the Decoded Message** $m^{-1}$	**0**	${IIF}_{\left(0,0\right)}$	${IIF}_{\left(1,0\right)}$
	**1**	${IIF}_{\left(0,1\right)}$	${IIF}_{\left(1,1\right)}$

**Table 2:** Incremental increase in information in the initial iterations for video steganogram No. 2.

Iteration	Level 1	Level 2	Level 3	Level 4	Level 5
1	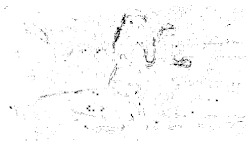	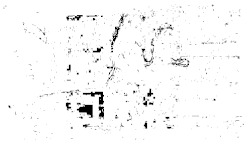	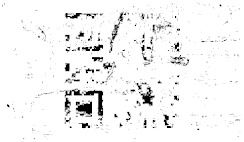	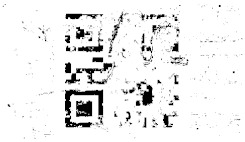	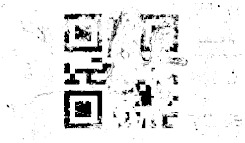
	IIF = 0.636	IIF = 0.672	IIF = 0.716	IIF = 0.772	IIF = 0.802
2	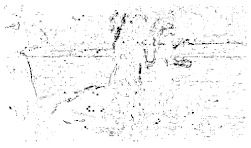	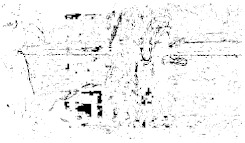	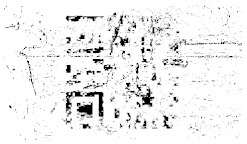	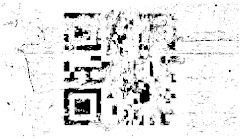	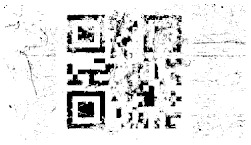
	IIF = 0.627	IIF = 0.669	IIF = 0.727	IIF = 0.796	IIF = 0.838
3	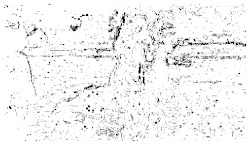	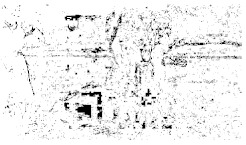	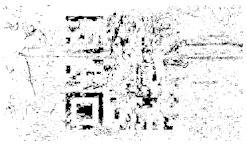	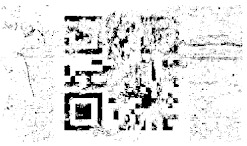	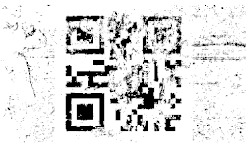
	IIF = 0.617	IIF = 0.667	IIF = 0.735	IIF = 0.812	IIF = 0.858
4	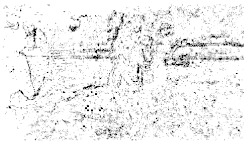	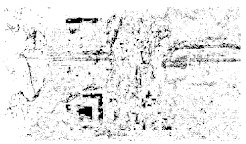	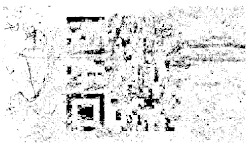	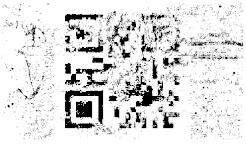	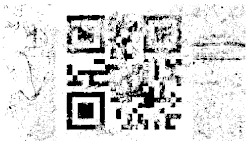
	IIF = 0.607	IIF = 0.662	IIF = 0.740	IIF = 0.821	IIF = 0.871
5	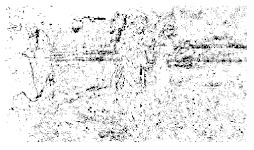	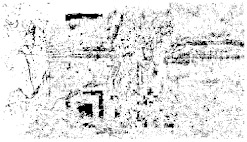	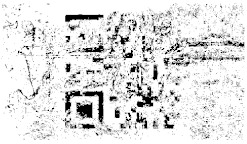	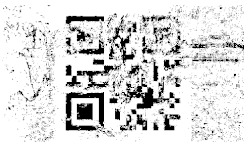	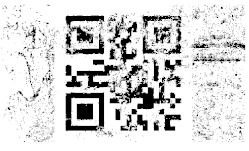
	IIF = 0.598	IIF = 0.661	IIF = 0.750	IIF = 0.840	IIF = 0.890
6	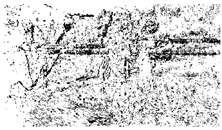	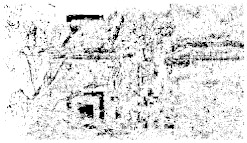	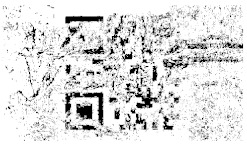	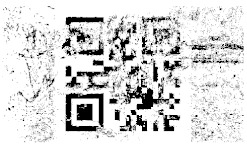	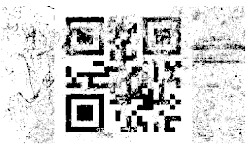
	IIF = 0.592	IIF = 0.659	IIF = 0.753	IIF = 0.845	IIF = 0.895
7	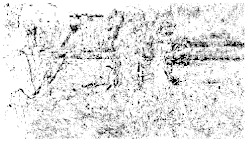	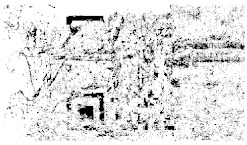	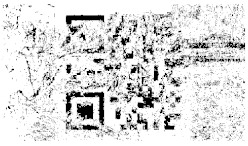	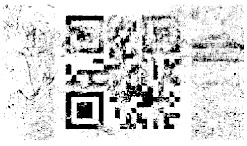	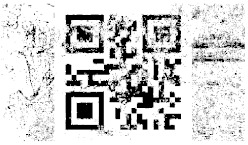
	IIF = 0.593	IIF = 0.664	IIF = 0.760	IIF = 0.852	IIF = 0.901
8	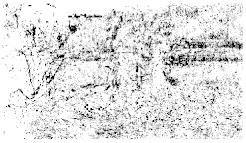	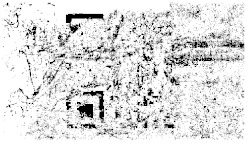	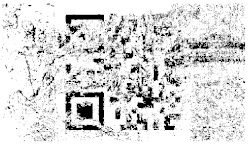	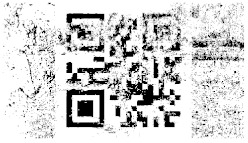	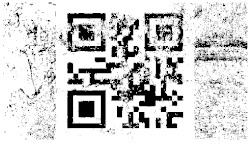
	IIF = 0.585	IIF = 0.661	IIF = 0.760	IIF = 0.854	IIF = 0.903
9	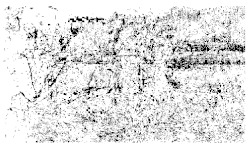	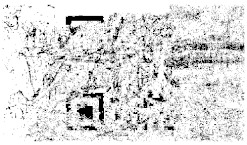	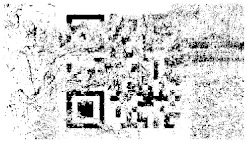	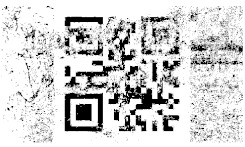	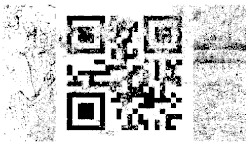
	IIF = 0.587	IIF = 0.664	IIF = 0.769	IIF = 0.864	IIF = 0.909
10	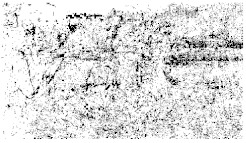	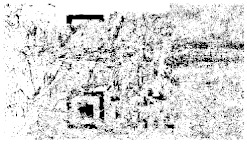	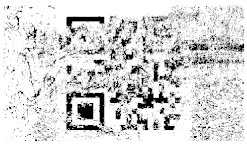	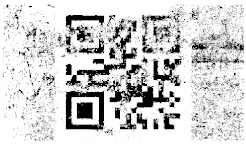	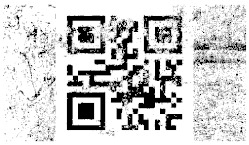
	IIF = 0.587	IIF = 0.669	IIF = 0.777	IIF = 0.873	IIF = 0.919

## Data Availability

The original contributions presented in this study are included in the article. Further inquiries can be directed to the corresponding author.
